# Goltz-Gorlin Syndrome: A Case Report and Literature Review

**DOI:** 10.7759/cureus.110528

**Published:** 2026-06-09

**Authors:** Sandra C Perez-Hernandez, Andrea Zaragoza-Navarro, Diana L Vazquez-Cantu, Claudia C Macías-Bueno, Georgina Sierra-Silva

**Affiliations:** 1 Dermatology, Instituto Dermatológico de Jalisco "Dr. José Barba Rubio", Zapopan, MEX; 2 Mucosal Diseases, Instituto Dermatológico de Jalisco "Dr. José Barba Rubio", Zapopan, MEX

**Keywords:** 5-fluorouracil, focal dermal hypoplasia, genodermatosis, goltz-gorlin syndrome, isotretinoin, papillomatous lesions

## Abstract

Goltz-Gorlin syndrome, also known as focal dermal hypoplasia (FDH), is an uncommon X-linked dominant mesoectodermal dysplasia that predominantly affects females, with most nonmosaic males presumed to be nonviable. The disorder arises from abnormal development of mesodermal and ectodermal tissues and manifests as a broad spectrum of multisystem abnormalities. It is characterized by distinctive cutaneous findings and extracutaneous involvement involving the ocular, dental, skeletal, GI, genitourinary, cardiovascular, and central nervous systems. We report the case of a 49-year-old woman who presented with multiple papillomatous lesions initially involving the oral cavity and progressively spreading to other areas. Physical examination revealed innumerable millimetric verruciform papules, as well as multiple soft, yellowish, smooth oval papules over a hyperpigmented macule distributed along Blaschko lines. Additional findings included telangiectasias, patchy alopecia, dental anomalies, strabismus, and syndactyly/ectrodactyly of the left hand and right foot. Histopathological examination of verruciform and yellow papules confirmed FDH. PCR testing for human papillomavirus from oral and heel lesions was negative. The diagnosis of Goltz-Gorlin syndrome was established based on clinical criteria and histopathological findings. Multidisciplinary management included dental excision of papillomas and treatment with topical 5-fluorouracil and oral isotretinoin, resulting in complete remission. To date, approximately 200-300 cases have been reported worldwide. A retrospective institutional review identified only two additional cases between 2010 and 2025, underscoring the rarity of this condition. This case highlights the importance of early recognition of Goltz-Gorlin syndrome through characteristic cutaneous and extracutaneous findings and suggests that combined topical 5-fluorouracil and oral isotretinoin may be an effective therapeutic option for extensive papillomatous lesions.

## Introduction

Goltz-Gorlin syndrome, also known as focal dermal hypoplasia (FDH; OMIM #305600), is a rare mesoectodermal dysplasia with X-linked dominant inheritance [1]. It primarily affects females and is considered lethal in most non-mosaic males. It was first described by Liebermann in 1935 as *atrophoderma linearis maculosa et papillomatosis congenitalis *and was later termed FDH by Goltz in 1962.

Goltz-Gorlin syndrome results from abnormal development of both mesodermal and ectodermal tissues, leading to a broad spectrum of multisystem manifestations [2]. The syndrome is characterized by distinctive cutaneous findings, including linear atrophic lesions distributed along Blaschko lines, papillomatous growths, and fat herniation. In addition, extracutaneous involvement is common and may affect the eyes, teeth, skeleton, GI tract, genitourinary tract, cardiovascular system, and central nervous system [3].

The molecular basis of FDH involves pathogenic variants in the *PORCN *gene, with more than 138 mutations reported to date. The *PORCN *gene (isoform D NM_203475.1), located on chromosome Xp11.23, encodes a 461-amino acid, 52-kDa endoplasmic reticulum protein and is the human homologue of the *Drosophila* polarity gene *porcupine *[4]. This protein functions as a putative O-acyltransferase involved in the palmitoylation and secretion of Wnt signaling proteins, a pathway that plays a crucial role in embryonic development, particularly in tissue patterning and skeletal formation [2,4].

Approximately 90% of reported female cases occur sporadically as a result of de novo mutations, whereas the remaining cases correspond to mosaic males [1,4]. Non-mosaic males are presumed to be nonviable. Survival in affected males has been explained by somatic mosaicism or, less commonly, by the presence of an additional X chromosome, as observed in Klinefelter syndrome. The wide phenotypic variability observed among affected females is attributed to functional mosaicism resulting from random X-chromosome inactivation, consistent with the Lyon hypothesis [4].

Although FDH is most commonly sporadic, familial cases with female-to-female transmission have been reported, as well as rare instances of father-to-daughter inheritance [3]. FDH is an exceptionally rare condition, with an estimated prevalence of less than 1 per 1,000,000 individuals. To date, approximately 200-300 cases have been reported in the literature worldwide [1]. After an extensive search of the PubMed and MEDLINE databases using the search terms “FDH”, “Goltz-Gorlin syndrome”, “focal dermal hypoplasia”, and “Mexico”, only three previously published case reports were identified, all involving female patients [5-7].

## Case presentation

A 49-year-old woman with no significant past medical history presented with multiple papillomatous lesions, which initially appeared in the mouth during her teenage years and have progressively spread to other areas of the body.

Dermatological examination revealed innumerable millimetric verruciform papules, either isolated or coalescing into well-demarcated plaques. In addition, multiple, yellowish, smooth, oval papules were observed over hyperpigmented macules distributed along Blaschko lines. Other cutaneous findings included telangiectasias and patchy alopecia. Extracutaneous manifestations were notable and included dental anomalies (malocclusion, enamel hypoplasia, and partial edentulism), strabismus, syndactyly, and ectrodactyly of the left hand and right foot (Figures 1-6).

**Figure 1:**
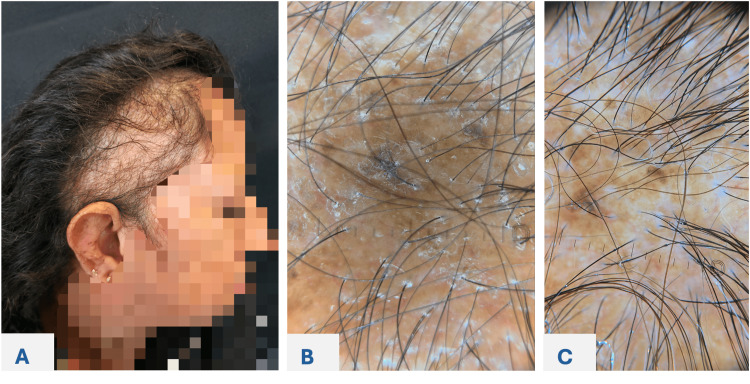
Trichoscopic findings of the scalp (A-C) Representative trichoscopic images demonstrating decreased hair density with focal atrichia and prominent hair shaft diameter diversity, characterized by the coexistence of terminal and miniaturized hairs. Follicular findings include multiple yellow dots and follicular keratotic plugging. Perifollicular changes are notable for scaling and erythema surrounding hair shafts. These features together suggest a mixed pattern of follicular miniaturization with superimposed perifollicular inflammatory changes.

**Figure 2:**
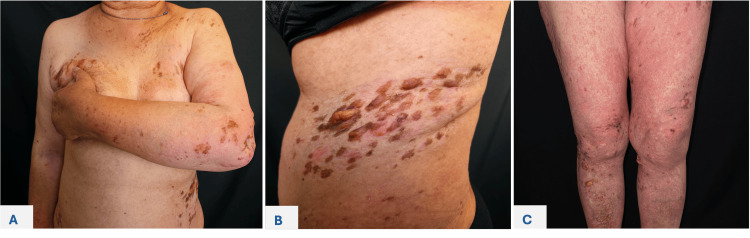
Cutaneous involvement (A-C) Clinical photographs showing asymmetric Blaschko-linear cutaneous lesions characterized by hyperpigmented macules and reticular atrophy, with some fat herniation changes involving the trunk and lower extremities.

**Figure 3:**
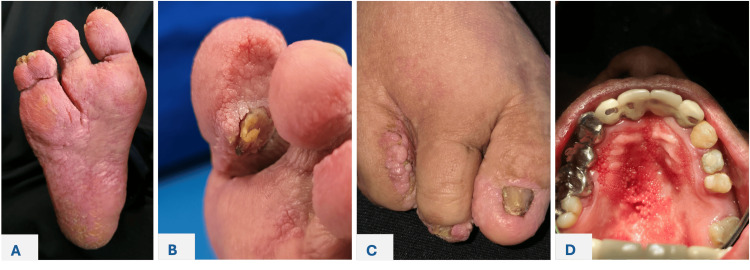
Papillomatous lesions (A-D) Clinical photographs showing multiple papillomatous lesions involving the digits, periungual areas, and oral mucosa, including the hard palate. Lesions display a clustered, verrucous, and raspberry-like appearance, consistent with papillomatous involvement.

**Figure 4:**
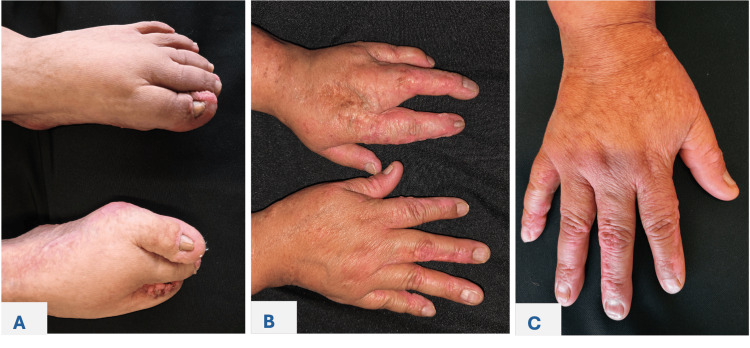
Skeletal and nail abnormalities (A) Clinical photograph of the feet showing digital abnormalities consistent with skeletal involvement. (B) Dorsal view of the hands demonstrating nail plate dysplasia and longitudinal ridging of the nail plates. (C) Close-up view highlighting nail dystrophy affecting multiple digits. Radiographic findings (A, B) included mild hypoplasia of the third metacarpal with fusion of the third proximal phalanx to the fourth and absence of the middle and distal phalanges of the third digit in the left hand. In the right foot, osseous fusion of the third and fourth metatarsals with complete absence of the phalanges of the third digit was reported.

**Figure 5:**
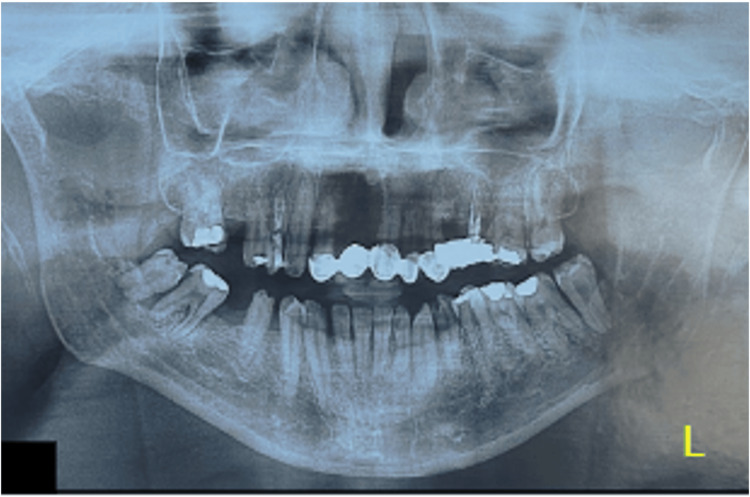
Panoramic radiograph Panoramic radiograph demonstrating multiple missing teeth, previous endodontic treatments, and generalized alveolar bone loss with a predominantly vertical pattern. Mesotaurodontism is observed in the mandibular third molars. Additionally, bilateral partial opacification of the maxillary sinuses is also noted, with increased radiopacity and reduced aeration.

**Figure 6:**
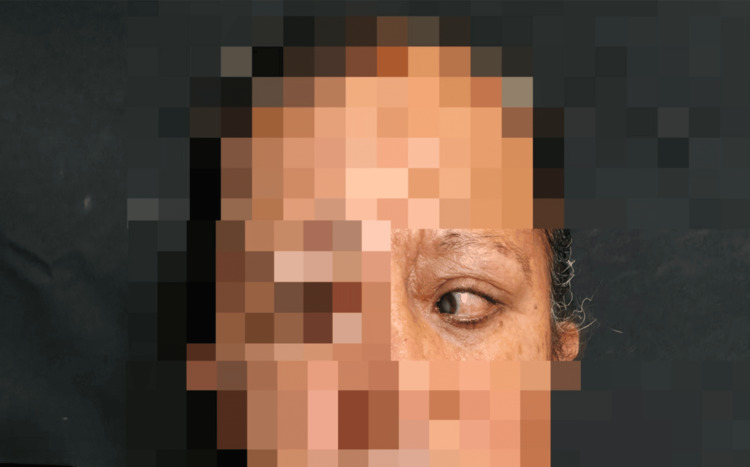
Ocular abnormalities Clinical photograph demonstrating left-sided ocular strabismus. The patient did not report visual impairment or functional visual limitation.

A detailed family pedigree was obtained. The patient was the product of a second pregnancy in a non-consanguineous family, with a total of seven live female siblings and one live male sibling, as well as two male stillbirths. No other affected family members were identified, supporting an X-linked disorder with a likely de novo mutation (Figure 7).

**Figure 7:**
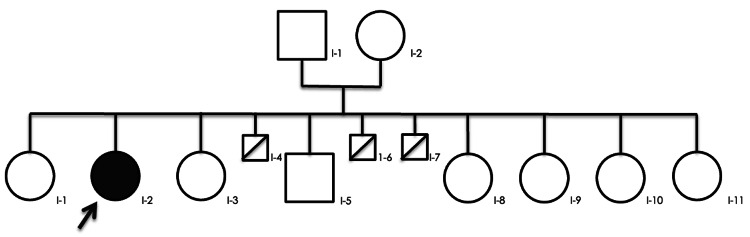
Family pedigree Family pedigree showing unaffected, non-consanguineous parents (I-1 and I-2) and multiple siblings. The proband (II-2), indicated by an arrow, is the only affected individual. Male deceased siblings are represented by squares crossed by a diagonal line. No other family members are affected. This original figure was created by the authors using Microsoft Word (Microsoft Corporation, Redmond, WA, USA).

Radiographic evaluation of the left hand showed five metacarpals in the left hand with mild hypoplasia of the third metacarpal; fusion of the third proximal phalanx with the fourth was observed, and the middle and distal phalanges of the third digit were absent. In the right foot, osseous fusion of the third and fourth metatarsals was identified, with complete absence of the phalanges of the third digit.

Histopathological examination of verruciform lesions from the oral cavity and heel showed mild to moderate acanthosis with focal papillomatosis and increased basal layer pigmentation. Biopsy of the yellowish papules was consistent with FDH (Figures 8, 9). PCR testing with genotyping for human papillomavirus (HPV) from both oral and heel papillomatous lesions was performed, yielding a negative result (Genes2Life. Ω-Plex VPH Cat. G2LOVPH-01).

**Figure 8:**
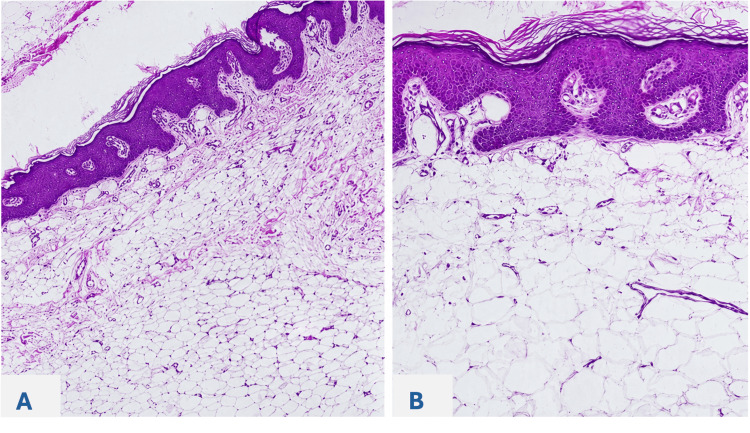
Histopathological features of FDH showing fat herniation (A) Low-power view demonstrating an acanthotic epidermis overlying a markedly thinned dermis, with predominance of subcutaneous tissue composed of mature adipocytes extending toward the superficial dermis (H&E, ×10). (B) Higher-magnification view highlighting the reduced dermal thickness and herniation of mature adipose tissue (H&E, ×40). FDH, focal dermal hypoplasia

**Figure 9:**
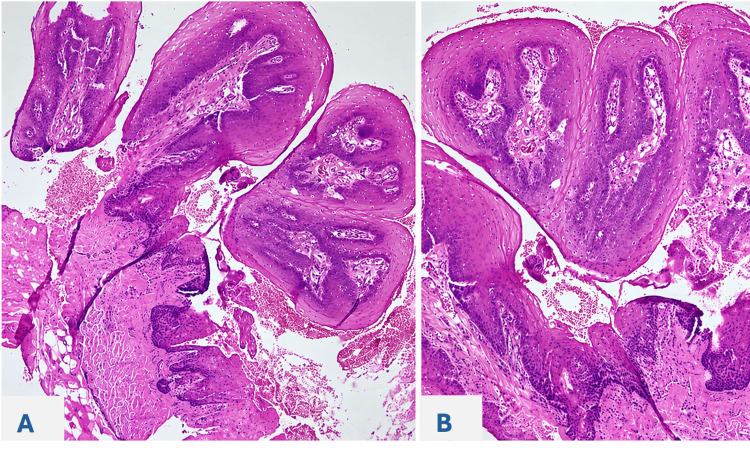
Histopathological features of a papillomatous lesion (A) Papillomatous epidermal proliferation with marked acanthosis and elongation of the rete ridges, without evidence of viral-induced keratinization (H&E, ×10). (B) Higher-magnification view showing dilated blood vessels within the underlying dermis, correlating with the papillomatous clinical morphology (H&E, inset ×40).

The diagnosis of Goltz-Gorlin syndrome, also known as FDH, was established based on the presence of three major and four minor diagnostic criteria. Given that the diagnosis of FDH is primarily clinical and histopathological and that the patient fulfilled the established diagnostic criteria, molecular testing for PORCN gene mutations was not pursued.

Due to the presence of extensive and symptomatic papillomatous lesions, a sequential therapeutic approach was implemented. Topical 5-fluorouracil 5% cream was applied once daily to the largest and most bulky lesions, resulting in an estimated reduction of approximately 80% in lesion size. Following this partial clinical response, oral isotretinoin was introduced as adjunctive therapy at a dose of 10 mg daily for three months, leading to marked clinical improvement and progressive flattening of the remaining papillomatous lesions (Figure 10). Baseline and monthly laboratory evaluations, including liver function tests and lipid profiles, were performed throughout the isotretinoin treatment period. No clinically significant laboratory abnormalities were detected, and the patient tolerated treatment well without treatment-related adverse events. Clinical improvement was maintained throughout a one-year follow-up period, with no evidence of lesion progression or recurrence.

**Figure 10:**
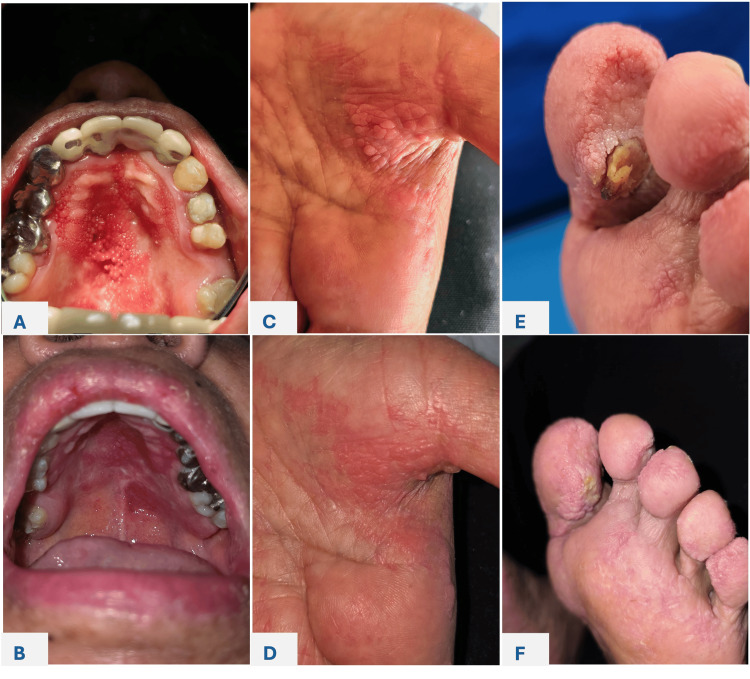
Papillomatous lesions before and after treatment (A-F) All images correspond to the same patient. (A, C, E) Baseline findings. (A) Multiple papules and papillomatous lesions involving the hard palate mucosa. (C) Clustered pink papules along the palmar fold with a linear distribution. (E) Periungual papillomatous lesion with hyperkeratosis and keratotic debris. (B, D, F) Clinical evolution after treatment with topical 5-fluorouracil and systemic isotretinoin. Marked reduction of the papillomatous component is observed in the oral mucosa (B), improvement of palmar lesions (D), and a significant decrease in the size of the periungual lesion (F).

## Discussion

Recognition of the phenotypic spectrum of Goltz-Gorlin syndrome is essential, as the diagnosis relies predominantly on the clinician’s ability to identify its characteristic clinical features. Due to its rarity and marked phenotypic variability, Goltz-Gorlin syndrome is frequently underrecognized or diagnosed late, particularly in adult patients.

Cutaneous manifestations represent the cornerstone of diagnosis and are present in the vast majority of affected individuals. The wide clinical variability observed in Goltz-Gorlin syndrome is explained by postzygotic somatic mutations in both sexes and by random X-chromosome inactivation in females, consistent with functional mosaicism [1]. This mechanism accounts for the asymmetric and Blaschko-linear distribution of lesions, as well as the heterogeneity in disease severity.

Although numerous cases of Goltz-Gorlin syndrome have been reported worldwide, fewer than 10 cases have been reported in the Mexican population (Tables 1, 2), underscoring the exceptional rarity of this condition and the limited regional clinical experience available. This limited number of reported cases highlights the importance of documenting additional patients to expand awareness and improve recognition of its diverse presentations.

**Table 1:** Clinical comparison of reported patients with Goltz-Gorlin syndrome from literature and institutional cases + = present; - = absent; NR = not reported

Clinical features	Corona-Guerra et al. (2018) [5]	Lopez-Porras et al. (2011) [6]	Contreras-Capetillo et al. (2014 [7]	Present case
Sex	Female	Female	Female	Female
Age at diagnosis	15 days	26 years	Infancy (evaluated at 5 years)	22 years
Current age	NR	Adult	5 years	49 years
Place of origin	Mexico	Mexico	Mexico	Guadalajara, Mexico
Gestational age	37 weeks	NR	Term	Term
Birth weight	2100 g	NR	Normal	NR
Apgar	8/9	NR	NR	NR
Mode of delivery	NR	NR	NR	Vaginal delivery
PORCN mutation	Negative/NR	Negative/NR	Positive	Not performed
Karyotype	46,XX	NR	46,XX	46,XX
Linear dermal hypoplasia	+	+	-	+
Blaschkoid lesions	+	+	+	+
Fat herniation	+	+	-	+
Papillomas	NR	+	NR	+
Telangiectasias	+	NR	NR	+
Alopecia/hypotrichosis	+	+	+	+
Nail dystrophy	NR	+	+	+
Syndactyly	+	+	+	+
Ectrodactyly	+	-	+	+
Polydactyly	-	+	-	-
Bone hypoplasia	+	+	+	+
Osteopathia striata	NR	NR	+	NR
Facial dysmorphism	+	+	+	+
Dental anomalies	NR	+	+	+
Cleft palate	+	+	-	-
Ear anomalies	+	+	+	+
Microphthalmia	-	NR	+	-
Coloboma	-	NR	+	-
Strabismus	NR	NR	NR	+
Other ocular findings	NR	NR	NR	NR
Renal anomalies	+	+	+	-
Cardiac anomalies	+	NR	NR	-
Neurodevelopmental delay	+	NR	-	-
Other anomalies	NR	NR	NR	NR

**Table 2:** Major and minor diagnostic criteria of patients with Goltz syndrome in reported and institutional patients + = present; - = absent; NR = not reported

Diagnostic criteria	Corona-Guerra et al. (2018) [5]	Lopez-Porras et al. (2011) [6]	Contreras-Capetillo et al. (2014 [7]	Present case
Major criteria				
Linear dermal hypoplasia	+	+	-	+
Fat herniation	+	+	-	+
Blaschkoid atrophic lesions	+	+	+	+
Mucocutaneous papillomas	NR	+	NR	+
Minor criteria				
Hypotrichosis/alopecia	+	+	+	+
Nail dystrophy	NR	+	+	+
Dental anomalies	NR	+	+	+
Facial dysmorphism	+	+	+	+
Ear anomalies	+	+	+	+
Skeletal anomalies	+	+	+	+
Syndactyly/ectrodactyly	+	+	+	+
Ocular anomalies	-	NR	+	+
Renal anomalies	+	+	+	-
Neurodevelopmental delay	+	NR	-	-

For appropriate clinical suspicion and diagnostic workup, it is crucial to recognize the broad spectrum of manifestations associated with Goltz-Gorlin syndrome. These can be categorized into cutaneous involvement, skeletal abnormalities, ocular findings, dental anomalies, nervous system and neurodevelopmental involvement, cardiovascular abnormalities, GI manifestations, renal and urinary tract abnormalities, and reproductive system involvement. Owing to the pleomorphic nature of the disease, not all manifestations are present in every patient, and clinical expression may vary significantly even within the same family.

Clinical manifestations

Skin Involvement

Cutaneous involvement is reported in more than 95% of patients and represents the most consistent and diagnostically relevant feature of Goltz-Gorlin syndrome. Skin findings may be evident at birth as erythematous, fragile, or atrophic areas and evolve over time into a heterogeneous pattern characterized by Blaschko-linear hypopigmentation or hyperpigmentation, reticular dermal atrophy, and telangiectasias. Lipomatous herniation and papillomatous lesions are commonly observed and are considered hallmark features of the syndrome [4]. More recently, progressive hyperpigmented freckling within hypopigmented areas has also been described.

Additional ectodermal manifestations include nail dystrophy, alopecia, and mucocutaneous papillomas. Nail abnormalities have been reported in up to 89% of cases and may include nail plate dysplasia, longitudinal ridging, V-shaped notching, anonychia, micronychia, and other dysplastic changes [3].

Hair involvement is also frequent and may present as diffuse or patchy alopecia with generalized hair thinning. Ultrastructural studies of hair shafts using scanning electron microscopy have demonstrated multiple abnormalities, including reduced hair shaft diameter, flattened cross-sectional morphology, trichorrhexis nodosa, pili torti, and pili trianguli et canaliculi, reflecting underlying defects in ectodermal development [3].

Raspberry-like papillomas are a characteristic feature of FDH and may involve multiple anatomical sites, occasionally leading to deformity and functional impairment. These lesions commonly affect the skin and mucous membranes, particularly the oral cavity, as well as the perioral region, esophagus, perianal area, genitalia (vulva and labia majora), and ocular regions. Importantly, papillomatous lesions in Goltz-Gorlin syndrome are noninfectious. In previously reported cases, fluorescent Q-PCR analysis has confirmed the absence of HPV types 6, 11, 16, and 18 in skin lesions, supporting a nonviral etiology [2,3].

Skeletal Disorders

Skeletal abnormalities represent the second most frequent extracutaneous manifestation of Goltz-Gorlin syndrome and are observed in approximately 60-70% of affected individuals. These defects are typically asymmetrical and highly variable in severity. Craniofacial anomalies may include facial asymmetry, ear deformities such as megalopinna and low-set ears, a characteristic pointed chin, and notched alae nasi. Less commonly, cleft lip and/or cleft palate have been reported [3].

Limb and orthopedic abnormalities are diverse and may include syndactyly, oligodactyly, ectrodactyly (classically described as “lobster-claw” deformity), polydactyly, and digital hypoplasia. Vertebral involvement has also been described and may manifest as scoliosis, kyphosis, vertebral body fusion, or limb length discrepancy. In approximately 20% of patients, longitudinal linear striations of the metaphyses of long bones, known as osteopathia striata, may be identified. Consequently, radiographic evaluation of the metaphyseal regions can be a valuable adjunct in the diagnostic assessment of Goltz-Gorlin syndrome [3,4].

Neurological and Neurodevelopmental Involvement

Neurological involvement in Goltz-Gorlin syndrome is variable and may include structural abnormalities such as myelomeningocele, Arnold-Chiari malformation, hydrocephalus, and seizure disorders [2,3]. Intellectual disability has been reported in approximately 15-18% of affected individuals. In addition, behavioral and emotional disturbances have been described, with withdrawn behavior reported by up to 65% of caregivers in some series. These findings underscore the importance of routine neurodevelopmental, cognitive, and behavioral assessment in patients with FDH to facilitate early intervention when indicated [3].

Ocular Abnormalities

Ocular involvement occurs in approximately 15% of patients with Goltz-Gorlin syndrome and is often asymmetrical. The most frequently reported abnormalities include microphthalmia and aniridia, followed by iris and chorioretinal colobomas and, less commonly, anophthalmia. Anterior segment anomalies, such as lacrimal duct abnormalities and corneal defects, are also considered classical manifestations. Recurrent papillomatous lesions arising from the conjunctiva and eyelid margins have additionally been described [2,3].

Dental Anomalies

Dental involvement is highly prevalent in Goltz-Gorlin syndrome and represents one of its most consistent extracutaneous features. Western cohort studies have shown that approximately 68% of patients exhibit hypodontia, microdontia, and vertical enamel grooving; 52% present with peg-shaped teeth; and up to 78% demonstrate enamel hypoplasia, with or without discoloration. Additional findings include gingivitis (reported in 25% of cases), dental caries, angular cheilitis (perleche), ridged gingiva, and malocclusion. Cleft lip and cleft palate have been reported in approximately 15% of patients [2-4].

Intraoral lipomas or papillomas may also be present without a specific site predilection. Dental malocclusion has been reported in up to 63% of individuals with Goltz-Gorlin syndrome and may be associated with speech difficulties or impaired mastication [2-4].

Cardiovascular Abnormalities

Cardiovascular involvement in Goltz-Gorlin syndrome is uncommon but may include congenital anomalies such as ventricular septal defects, hypoplasia of the pulmonary arteries or veins, mediastinal dextroposition, and, rarely, ectopia cordis [2].

GI Involvement

GI manifestations are less frequent and often related to defects in the abdominal wall. Hernias may occur at sites of cutaneous hypoplasia, and diaphragmatic hernia has also been reported in association with Goltz-Gorlin syndrome [2].

Kidneys and Urinary System

Renal and urinary tract involvement may predispose patients to recurrent urinary tract infections and vesicoureteral reflux. Reported abnormalities include renal hypoplasia, unilateral renal agenesis, cystic renal dysplasia, hydronephrosis, and fused or horseshoe kidneys [3].

Reproductive System

Reproductive system anomalies predominantly affect females, who represent approximately 90% of reported cases. Uterus bicornis is the most frequently described abnormality, followed by a septate hymen, athelia, and mammary hypoplasia. In males, genital abnormalities such as bilateral cryptorchidism have also been reported, although these cases are rare due to the typically lethal nature of non-mosaic male disease [3,4].

Diagnostic criteria

The diagnostic criteria proposed by Bostwick et al. have been widely accepted as a reliable framework for the diagnosis of Goltz-Gorlin syndrome [8]. With increasing clinical experience and the accumulation of reported cases, these criteria have been refined and expanded, leading to the recognition that molecular genetic testing is not mandatory for diagnosis in patients with a characteristic phenotype. Instead, FDH can be diagnosed clinically based on the presence of three or more characteristic cutaneous findings in combination with at least one typical limb malformation [3].

Major diagnostic criteria predominantly reflect ectodermal and skeletal involvement and constitute the core features of the syndrome. Minor criteria include additional ectodermal manifestations, as well as dental, hair, and ocular abnormalities that further support the diagnosis (Figure 11).

**Figure 11:**
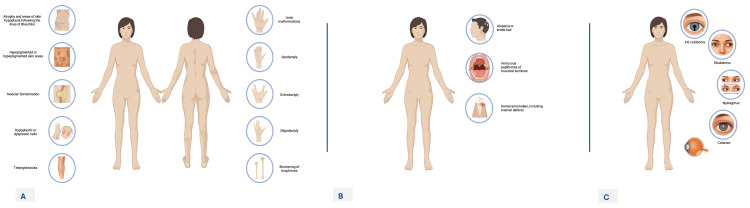
Diagnostic criteria (A) Major criteria. (B) Minor criteria. (C) Other manifestations. FDH can be diagnosed clinically based on the presence of three or more characteristic cutaneous findings in combination with at least one typical limb malformation [3]. FDH, focal dermal hypoplasia This original figure was created by the authors using BioRender (BioRender.com, Toronto, ON, Canada) without the use of AI.

Taken together, these criteria emphasize that the diagnosis of Goltz-Gorlin syndrome is primarily clinical, underscoring the importance of thorough phenotypic evaluation and careful recognition of its multisystem manifestations, particularly in settings where access to molecular testing may be limited.

Histopathological findings

Histopathological examination of the skin biopsy revealed features characteristic of FDH. The dermis was markedly thinned and atrophic, with focal herniation of mature adipose tissue extending into the superficial dermis. Prominent papillomatous changes were also observed, consisting of epidermal hyperplasia accompanied by hyperkeratosis and acanthosis.

These histological findings are believed to reflect an underlying developmental defect of mesodermal derivatives and are considered hallmark features of FDH. Histopathological evaluation can be particularly valuable in cases with subtle or atypical cutaneous manifestations, as it provides supportive evidence when clinical findings alone are insufficient to establish the diagnosis.

Microscopic examination consistently demonstrated reduced dermal thickness with sparse and loosely arranged collagen bundles, resulting in a hypocellular dermis. The overlying epidermis was relatively preserved despite its reduced thickness. Elastic fibers were markedly decreased, a finding best appreciated with special histochemical stains, and this reduction likely contributes to the increased fragility and atrophic appearance of the affected skin [3,4].

Papillomatous lesions, which are frequently observed in FDH, demonstrated prominent papillomatosis with epidermal hyperplasia, hyperkeratosis, and acanthosis. Increased vascularity within the papillary dermis was also identified, correlating with the friable, raspberry-like appearance observed on clinical examination. No viral cytopathic changes were present, supporting a noninfectious etiology and suggesting that these papillomatous lesions represent an intrinsic manifestation of the disease rather than a secondary process [3,4].

Overall, the combination of dermal hypoplasia with adipose tissue replacement and characteristic papillomatous epidermal changes constitutes the histopathological hallmark of FDH. These findings complement the clinical diagnostic criteria and provide additional support for diagnosis, particularly in patients with incomplete or variable phenotypic expression [3,4].

Treatment

Management of Goltz-Gorlin syndrome is primarily supportive and should be individualized according to the patient’s specific clinical manifestations. Given the multisystem nature of the disorder, treatment strategies are aimed at improving function, cosmetic appearance, and overall quality of life. General management measures include genetic counseling as a form of primary prevention, reconstructive surgery to correct functional defects, and orthopedic interventions when indicated [2-4].

Cutaneous management is largely focused on the treatment of papillomatous lesions and the alleviation of associated symptoms such as pruritus and erythema. Previous reports have described the use of a flashlamp-pumped pulsed dye laser to reduce erythema and flatten lesions, as well as cryotherapy for the treatment of large or symptomatic papillomas involving the trunk and extremities [4]. However, standardized therapeutic protocols for extensive or recurrent papillomatous lesions are lacking, and clinical responses remain variable.

In the present case, an alternative therapeutic strategy was implemented for the management of extensive and symptomatic papillomatous lesions. Topical 5-fluorouracil was initially applied to the largest lesions, resulting in an estimated reduction of approximately 80% in lesion size. Following this partial response, oral isotretinoin was introduced as adjunctive therapy, leading to further clinical improvement and progressive flattening of the remaining papillomatous lesions. To the best of our knowledge, the sequential use of topical 5-fluorouracil for bulky papillomatous lesions followed by systemic isotretinoin as complementary therapy has not been previously reported in patients with FDH.

Management of extracutaneous manifestations remains challenging, particularly in individuals with limb malformations, orofacial clefts, or significant ocular involvement, and often requires a multidisciplinary approach. Despite these challenges, patients with FDH generally have a normal life expectancy and, with appropriate long-term multidisciplinary care, can achieve good functional outcomes and maintain a satisfactory quality of life [4].

## Conclusions

Goltz-Gorlin syndrome is a rare mesoectodermal dysplasia with X-linked dominant inheritance and variable clinical expression that may hamper and delay diagnosis, particularly in adult patients. In Mexico, only a few cases have been reported over the past 15 years, highlighting the importance of recognizing its characteristic cutaneous and extracutaneous manifestations to facilitate timely diagnosis. This case emphasizes the importance of clinicopathologic correlation as a key tool for confirming the diagnosis in the absence of genetic testing, as well as the value of recognizing the major and minor clinical criteria that support the diagnosis.

The combined use of topical and systemic therapies, such as 5-fluorouracil cream and oral isotretinoin, may represent a promising therapeutic approach for the management of extensive papillomatous lesions when integrated into a multidisciplinary treatment strategy. However, further studies are needed to better define its potential role in patients with FDH.
